# Smartwatch-Based Ecological Momentary Assessment for High-Temporal-Density, Longitudinal Measurement of Alcohol Use (AlcoWatch): Feasibility Evaluation

**DOI:** 10.2196/63184

**Published:** 2025-03-25

**Authors:** Chris Stone, Sally Adams, Robyn E Wootton, Andy Skinner

**Affiliations:** 1 Integrative Cancer Epidemiology Programme Bristol Medical School University of Bristol Bristol United Kingdom; 2 School of Psychological Science University of Bristol Bristol United Kingdom; 3 School of Psychology University of Birmingham Birmingham United Kingdom; 4 Nic Waals Institute Lovisenberg Diaconal Hospital Oslo Norway; 5 Medical Research Council Integrative Epidemiology Unit Bristol Medical School University of Bristol Bristol United Kingdom; 6 PsychGen Centre of Genetic Epidemiology and Mental Health Norwegian Institute of Public Health Oslo Norway

**Keywords:** smartwatch, ecological momentary assessment, μEMA, alcohol, ALSPAC

## Abstract

**Background:**

Ecological momentary assessment methods have recently been adapted for use on smartwatches. One particular class of these methods, developed to minimize participant burden and maximize engagement and compliance, is referred to as microinteraction-based ecological momentary assessment (μEMA).

**Objective:**

This study explores the feasibility of using these smartwatch-based μEMA methods to capture longitudinal, high-temporal-density self-report data about alcohol consumption in a nonclinical population selected to represent high- and low-socioeconomic position (SEP) groups.

**Methods:**

A total of 32 participants from the Avon Longitudinal Study of Parents and Children (13 high and 19 low SEP) wore a smartwatch running a custom-developed μEMA app for 3 months between October 2019 and June 2020. Every day over a 12-week period, participants were asked 5 times a day about any alcoholic drinks they had consumed in the previous 2 hours, and the context in which they were consumed. They were also asked if they had missed recording any alcoholic drinks the day before. As a comparison, participants also completed fortnightly online diaries of alcohol consumed using the Timeline Followback (TLFB) method. At the end of the study, participants completed a semistructured interview about their experiences.

**Results:**

The compliance rate for all participants who started the study for the smartwatch μEMA method decreased from around 70% in week 1 to 45% in week 12, compared with the online TLFB method which was flatter at around 50% over the 12 weeks. The compliance for all participants still active for the smartwatch μEMA method was much flatter, around 70% for the whole 12 weeks, while for the online TLFB method, it varied between 50% and 80% over the same period. The completion rate for the smartwatch μEMA method varied around 80% across the 12 weeks. Within high- and low-SEP groups there was considerable variation in compliance and completion at each week of the study for both methods. However, almost all point estimates for both smartwatch μEMA and online TLFB indicated lower levels of engagement for low-SEP participants. All participants scored “experiences of using” the 2 methods equally highly, with “willingness to use again” slightly higher for smartwatch μEMA.

**Conclusions:**

Our findings demonstrate the acceptability and potential utility of smartwatch μEMA methods for capturing data on alcohol consumption. These methods have the benefits of capturing higher-temporal-density longitudinal data on alcohol consumption, promoting greater participant engagement with less missing data, and potentially being less susceptible to recall errors than established methods such as TLFB. Future studies should explore the factors impacting participant attrition (the biggest reason for reduced engagement), latency issues, and the validity of alcohol data captured with these methods. The consistent pattern of lower engagement among low-SEP participants than high-SEP participants indicates that further work is warranted to explore the impact and causes of these differences.

## Introduction

Ecological momentary assessment (EMA) refers to a class of methods in which people are asked to answer questions about specific aspects of their health, feelings, and behaviors as they go about their normal lives. As individuals typically complete these assessments throughout the day, rather than completing a survey and having to remember details from days or weeks ago, recall errors are significantly reduced [1]. One domain in which EMA methods have been used extensively is the study of modifiable health behaviors, including physical activity, diet, tobacco smoking, and alcohol consumption [2]. The benefits of EMA methods are of particular interest for the measurement of alcohol consumption. Commonly used questionnaires and diary-based methods are known to suffer from systematic biases toward underreporting, capturing somewhere between 20% and 60% of the true level of consumption [3,4], with retrospective recall bias being a major factor [5]. Furthermore, recent studies have highlighted the importance of both longitudinal measures of alcohol consumption to help us explore relationships between alcohol consumption and conditions such as depression [6], and high-temporal-resolution measures of alcohol consumption to enable exploration of factors such as subjective responses (eg, feeling of stimulation, feeling of sedation, liking the effects of alcohol, wanting more alcohol) in heavy drinkers in natural environments [7]. There is, therefore, a need for EMA methods that can be used to capture high-temporal-density measures of alcohol consumption over extended periods.

In response to the increased adoption of mobile and wearable technologies, EMA methods have been adapted for use on smartphones, and more recently, smartwatches. For smartwatch-based EMA methods, a particular area of focus has been reducing participant burden, so that even more frequent sampling over longer periods can be tolerated. Smartwatches are worn on the wrist so they are never beyond reach, which reduces the time to access the device and respond to prompts. Further, because they are worn against the body, they enable the use of potentially more discrete haptic prompts for responses. One smartwatch EMA method aiming to minimize participant burden is microinteraction-based EMA (μEMA), which uses simple, brief questions with a limited set of answers that can be responded to with a single tap multiple times a day [8]. When compared with smartphone-based EMA over a 4-week period, smartwatch-based μEMA had better performance in terms of compliance rates (defined as the percentage of all scheduled prompts to which participants responded, regardless of the success of prompt delivery: 82% vs 64%) and completion rates (defined as the percentage of scheduled prompts actually delivered to the participant to which they responded: 92% vs 67%) [8].

Having demonstrated high levels of engagement in these initial 4-week explorations of smartwatch-based μEMA, the approach was subsequently used to capture self-report measures over longer periods in various settings. Beukenhorst and colleagues [9] explored the use of smartwatch-based μEMA methods in participants with osteoarthritis of the knee to capture self-report measures of pain and quality of life 4 or 5 times a day over 3 months. The compliance rate across all participants ranged from around 85% at the beginning of the study to around 30% at the end of the 3 months. The authors suggested the reduction over time was largely a result of participant attrition, possibly driven by technical factors including poor watch battery life. Considering just active participants, compliance remained above 75% throughout the 3 months. Ponnada and colleagues [10] explored using the same smartwatch-based μEMA methods for capturing self-report data of physical activity and its effect in young adults over an even longer time frame of 12 months. Preliminary findings at 6 months (when the authors published interim results of their study) indicated an overall compliance of rate 67% and a completion rate of 79% [10]. These findings suggest that while it is necessary to be mindful of technical issues that can lead to attrition, smartwatch-based μEMA appears to be a viable method for collecting high-temporal-resolution self-report data over extended time frames.

In terms of the effect of sample characteristics on EMA study compliance, several recent studies have reported mixed findings for age and sex [11-13]. One sample characteristic not explored as extensively is socioeconomic position (SEP). For studies of alcohol use, SEP is of particular importance as there are known to be complex relationships between SEP, alcohol consumption, and health outcomes (the “alcohol harm paradox”), in which individuals are more likely to have drunk in the past week and drank 6 units of alcohol or more in 1 drinking episode if they are a high-income earning managerial/professional worker [14,15], but individuals with lower individual or neighborhood SEP have an increased susceptibility to the negative and harmful health effects of alcohol consumption [16,17]. Understanding if SEP has a systematic effect on engagement with any new methods measuring alcohol use is therefore clearly important.

The objective of this study was to explore the feasibility of using smartwatch-based μEMA methods for the capture of high-resolution self-reported data about alcohol use over extended periods in high- and low-SEP groups. We asked participants to use a smartwatch-based μEMA system to record self-reported data about alcohol use and the context in which alcohol was consumed 5 times a day over a 3-month period. For comparison, we also asked all participants to record their alcohol use every 2 weeks using an online version of one of the currently most established methods for capturing data on alcohol consumption, the Timeline Followback (TLFB) method. We report engagement metrics (compliance and completion) for both smartwatch μEMA and TLFB methods, qualitative experiences of using both methods captured in interviews at the end of the study, and some initial findings comparing the levels of alcohol recorded by the 2 methods at the level of individual participants. Finally, in line with recommendations from a recent evaluation of pressing issues in EMA studies [18], we also report the characteristics of missing data for the smartwatch μEMA for all participants and high- and low-SEP groups, to explore whether SEP impacts the nature of missing data in a systematic manner.

## Methods

### Study Overview

**Figure 1:**
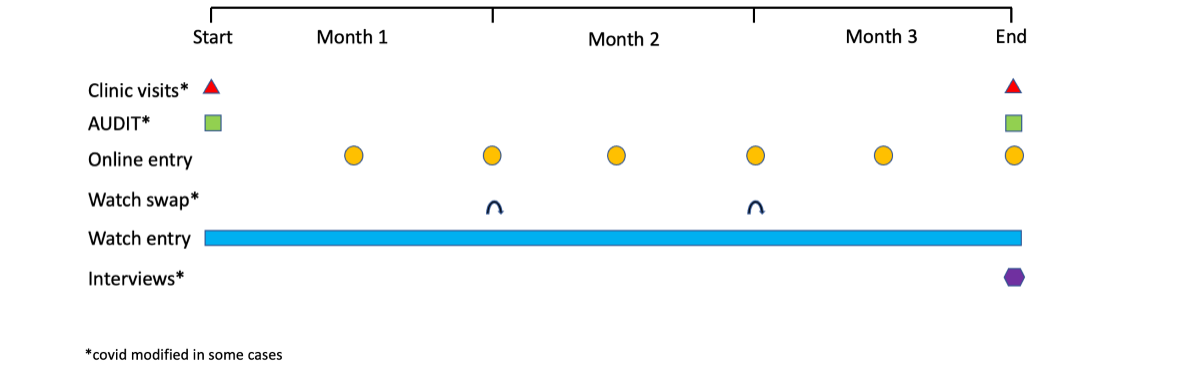
Overview of study elements with timings.

The design and analyses of the study followed those detailed in the preregistered study protocol [19]. Any deviations from the study protocol are detailed in the following sections. An overview of the study elements, with their timings throughout the study, is shown in Figure 1. Data collection happened between September 2019 and June 2020.

### Study/Clinical Setting

This study was embedded within the Avon Longitudinal Study of Parents and Children (ALSPAC), a longitudinal birth-cohort study. During phase I of ALSPAC enrollment, 14,541 pregnancies in the former Avon Health Authority in the southwest of England with expected dates of delivery between April 1, 1991, and December 31, 1992, were recruited. Of these initial pregnancies, there was a total of 14,676 fetuses, resulting in 14,062 live births and 13,988 children who were alive at 1 year of age. A further 906 pregnancies were recruited during phases II, III, and IV, respectively, resulting in an additional 913 children being enrolled. The total sample size is 15,447 pregnancies, of which 14,901 were alive at 1 year of age.

Further details on the cohort profile, representativeness, and phases of recruitment are described in 3 cohort-profile papers [20-22].

The ALSPAC study website provides details on all available data through a fully searchable data dictionary and variable search tool [23]. Informed consent for data collected via questionnaires and clinics was obtained from participants in accordance with the recommendations of the ALSPAC Ethics and Law Committee at the time.

In this study, data were captured using smartwatch-based μEMA techniques as participants went about their normal daily lives and through online TLFB methods periodically throughout the study. An initial session was held at the ALSPAC premises in the Bristol Medical School, during which participants were briefed, provided consent, and completed questionnaires. A final session was also held there, where participants were debriefed, completed a qualitative interview about their experiences during the study, and returned equipment.

Study data for the TLFB method were collected and managed using REDCap, a secure, web-based electronic data capture platform hosted at the University of Bristol. REDCap (Research Electronic Data Capture) is designed to support data capture for research studies [24].

### Ethical Considerations

#### Ethics Review

As this research involved human participants, ethics approval was obtained from the ALSPAC Ethics and Law Committee, which reviewed the study and granted formal approval (reference code 83643) in accordance with the University of Bristol institutional guidelines.

#### Informed Consent

Prospective participants received an information sheet before the initial study session, explaining the study’s nature, purpose, and risks, and were given the opportunity to ask investigators any questions before deciding to participate. Upon arrival at the initial session, participants could review the information sheet again and ask further questions. Informed consent for data collection was then obtained through a written consent form, following the recommendations of the ALSPAC Ethics and Law Committee. Participants were also informed that they could withdraw from the study at any time without providing a reason.

#### Privacy and Confidentiality

The data obtained from participants have been anonymized to ensure they are not identifiable. No individual participant can be identified from this manuscript or its multimedia appendices.

#### Compensation

Participants were reimbursed £15 (US $19.41) per month for their time and expenses, with a bonus of £15 (US $19.41) for completing all 3 months. Thus, those who completed the full study received a total of £60 (US $78 at the time of the study).

### Participants

Participants were drawn from the first generation of children born in the ALSPAC study (ALSPAC-G1) and were selectively recruited by SEP based on maternal highest educational attainment. Those whose mothers had a degree or higher were assigned to the high-SEP group, while those whose mothers had less than a degree were assigned to the low-SEP group.

The inclusion criteria were as follows:

- Consume at least 6 units of alcohol per week.
- Be able to wear and use the smartwatch from 12 PM to 10 PM (the timings of questions in the current version of the smartwatch system cannot be altered to accommodate shift work, etc)
- Have access to the internet to complete the TLFB assessments.

Exclusion criteria (standard exclusion criteria used in alcohol studies to prevent harm during pregnancy and to those at risk of addiction) were as follows:

- Pregnant or breastfeeding (self-reported). Note that if a participant becomes pregnant during the study, they must withdraw from the study at this point.
- History of alcohol or drug addiction (self-reported).
- Strong familial history of alcoholism defined as 1 or more immediate relatives (parent, sibling) or more than 1 other relative (eg, cousin, grandparent; self-reported).

### Clinic Visits

All participants began the study with a visit to an ALSPAC clinic. In this session (approximately 1 hour in duration) participants

- were given details of the study and provided written consent;
- were given the smartwatch and shown how to use the μEMA application and maintain the smartwatch (including instructions on keeping it charged, not getting it wet, and removing it before contact sports);
- were given instructions on how to use the online TLFB diary, provided with log-on details, and asked about any events (eg, birthdays, nights out) that should be entered into the diary as reminders;
- were provided with contact details of whom to call if they encountered any issues during the study.

Participants completed a second clinic visit at the end of the study. In this session (approximately 30 minutes) participants

- returned the smartwatch;
- completed a brief semistructured interview about their experiences using the smartwatch and online TLFB diary led by the researcher;
- provided written final consent; and
- were given their cash reimbursement.

A total of 13 participants (8 low SEP and 5 high SEP) completed the second clinic visit in person.

As a result of COVID-19 restrictions, the remaining 19 participants (11 low SEP and 8 high SEP) completed their second clinic visit remotely. In these remote sessions, ALSPAC researchers conducted telephone interviews, and smartwatch collection was arranged via courier.

### Smartwatch Data Collection

We developed a bespoke μEMA smartwatch application incorporating elements proposed by Intille et al [8] to reduce participant burden. These included using haptic rather than audible prompts to minimize disruption and designing brief questions with a limited set of single-tap responses to reduce effort.

To optimize our app design, we collaborated with the ALSPAC Original Cohort Advisory Panel, a committee representing the diverse backgrounds and perspectives of ALSPAC participants. Using a think-aloud approach, panel members walked through the μEMA application, identifying issues related to visual design, wording, and navigation. Several common themes emerged, leading to the following changes in the final μEMA application design:

- The introduction of the “My usual” shortcut to speed up the recording of alcoholic drinks that are the same type, quantity, and context.
- The introduction of the “Back” option so participants could go back and correct previous entries in the current epoch.

Participants received a TicWatch C2 (Mobvoi Information Technology Company Limited) smartwatch running Android Wear 2.6 with our μEMA application preloaded.

At 5 time points each day (2 PM, 4 PM, 6 PM, 8 PM, and 10 PM), participants were asked about any alcoholic drinks consumed in the past 2 hours and the context of these drinking events. At each time point, they were presented with the set of questions and response options listed in Textbox 1.

**Figure 2:**
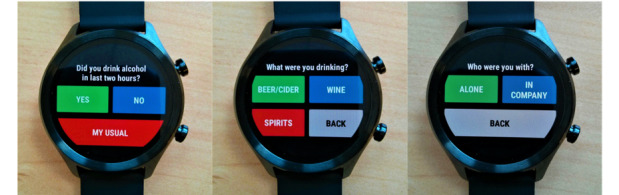
Example smartwatch microinteraction-based ecological momentary assessment (μEMA) application questions.

Examples of these questions as rendered in our μEMA application are shown in Figure 2, with the full question sequence illustrated in Multimedia Appendix 1.

**Questions and options presented to participants.**

- Question 1: Did you drink alcohol in the last 2 hours? Response options [Yes | No | My Usual].
- Question 2: What were you drinking? Response options [Beer/cider | Wine | Spirits ].
- Question 3: What size was your drink? Response options: Beer/cider: [Half a pint | 330 bottle | Pint], Wine: [125 mL | 175 mL |250 mL], Spirits: [Single | Double | Free pour].
- Question 4: Who were you with? Response options [Alone | In company].
- Question 5: Where were you? Response options [At home | Elsewhere].
- Question 6 Any more alcoholic drinks to be recorded? Response options [Yes | No | My Usual].

“Usual”: On the first day of the study, participants were given the option to set up a “My Usual” shortcut to simplify data entry for their most frequently consumed alcoholic drink. If they opted in, they answered questions 2-5 to specify their usual drink. Thereafter, selecting “My Usual” automatically recorded those drink details for that time point.

Each day at noon, participants were asked if they had missed recording any drinks the previous day. If they responded “Yes,” they were prompted to report those drinks using the same set of questions.

Captured data were not visible to participants and were stored locally on the smartwatch rather than uploaded to a cloud-based platform to minimize the risk of personal data loss. As a result of the lack of remote monitoring, smartwatches were swapped at the end of months 1 and 2 to mitigate the risk of undetected technical issues disrupting data collection. However, COVID-19 restrictions prevented this for 15 participants (7 low SEP and 8 high SEP), who were instead instructed to continue using their current smartwatch until the study’s conclusion.

### Online Data Collection

The TLFB method uses a calendar prepopulated with participant-defined diary entries (eg, birthdays, theatre visits, nights out) to aid in recall of alcohol consumption. Online self-administration of TLFB has been validated [25], and previous studies suggest that a 14-day recall period optimally balances data quality and participant burden [26]. Therefore, participants completed an online TLFB every 14 days, recording their alcohol consumption over the previous 2 weeks.

We implemented TLFB using REDCap [24], hosted on the ALSPAC application platform. Participants had a 5-day window to complete each 2-week TLFB entry and received SMS text message reminders at the start of this period. In each entry, participants recorded the number of alcohol units consumed each day. As data captured in the smartwatch μEMA application were not visible to participants, they could not use it to complete TLFB entries. In line with standard TLFB practice, no context questions were asked. The format of the TLFB diary entry screen is shown in Multimedia Appendix 2.

### Interviews

In the second clinic session at the end of the study (conducted either in person or remotely by telephone), each participant completed a semistructured interview exploring their experiences during the study.

For both smartwatch μEMA and online TLFB methods, participants were asked:

Question 1/6: Overall, how would you rate your experience of using the smartwatch/online system during the study, on a scale from 1 (I did not like it at all) to 10 (I really liked it)?

Question 2/7: If you were asked to use the smartwatch/online system again in another study, how likely would you be to say yes, on a scale from 1 (I would not want to use it again) to 10 (I would really like to use it again)?

Question 3/8: If you agreed to use the smartwatch/online system again in another study, what is the maximum length of time you would be willing to use it for?

Question 4/9: What things did you like about using the smartwatch/online system?

Question 5/10: What things did you dislike about using the smartwatch/online system?

Participants were asked additional questions specifically about their experiences using the smartwatch μEMA system:

Question 11: How did you feel about having to charge the smartwatch every night?

Question 12: Did you use any of the other functions on the smartwatch during the study (eg, step counter)?

Question 13: Did you pair the smartwatch with any smartphones during the study?

Participants were also asked about their attitudes toward future developments of the smartwatch μEMA system:

Question 14: How would you feel if the smartwatch system used data from its motion sensors to work out when the best time is to ask you the questions (eg, so it does not ask you questions when you are driving)?

Question 15: How would you feel if the smartwatch system used data from its global positioning system (GPS) location sensor (which tells the watch where the person is) to work out when the best time is to ask you the questions (eg, so it does not ask you questions when you are at work)?

### Engagement Measures

In reporting engagement, we follow the approach used by Intille and colleagues [8]. We report compliance rates, defined as the proportion of scheduled questions answered by participants, both for all participants who started the study and for those still active in the study. Participants are considered active until they make their final response and drop out.

In addition, for smartwatch μEMA, we report the completion rate, defined as the proportion of questions delivered to active participants by the μEMA application that they respond to. This helps distinguish whether nonresponses result from factors preventing question delivery (eg, the smartwatch being switched off or the battery being flat) or from participants’ propensity and ability to respond to delivered questions. The completion rate is not reported for online TLFB, as questions were not actively presented to participants, meaning there were no prompts that could be delivered or missed.

For all engagement measures, we report data for all participants as well as separately for the low- and high-SEP groups.

### Missing Data

We examined whether prompts were more likely to be missed at certain times of the day by comparing missingness rates across 2-hour epochs. Similarly, we assessed whether prompts were more likely to be missed on specific days of the week using the same approach. These analyses were conducted for all participants as well as separately for the low- and high-SEP groups.

### Comparison of Levels of Alcohol Consumption Recorded Using the Two Methods

To provide an initial indication of whether μEMA might capture higher levels of alcohol consumption than TLFB, we calculated the total number of units consumed over the 12-week study period for each participant, using only weeks where data were available from both methods. Within the high- and low-SEP groups, we compared the number of participants whose recorded consumption was higher in one method than the other. Note that this was an exploratory analysis and not preplanned.

## Results

### Participants

Our original aim was to recruit 40 participants (20 low SEP and 20 high SEP). However, recruitment had to be halted prematurely in March 2020 due to COVID-19 restrictions, by which point 32 participants had been recruited. Of these, 13 were high SEP (mean age 27 years, range 26-28 years; 7, 54%, females) and 19 were low SEP (mean age 28 years, range 26-28 years; 13, 68%, females).

### Engagement

**Figure 3:**
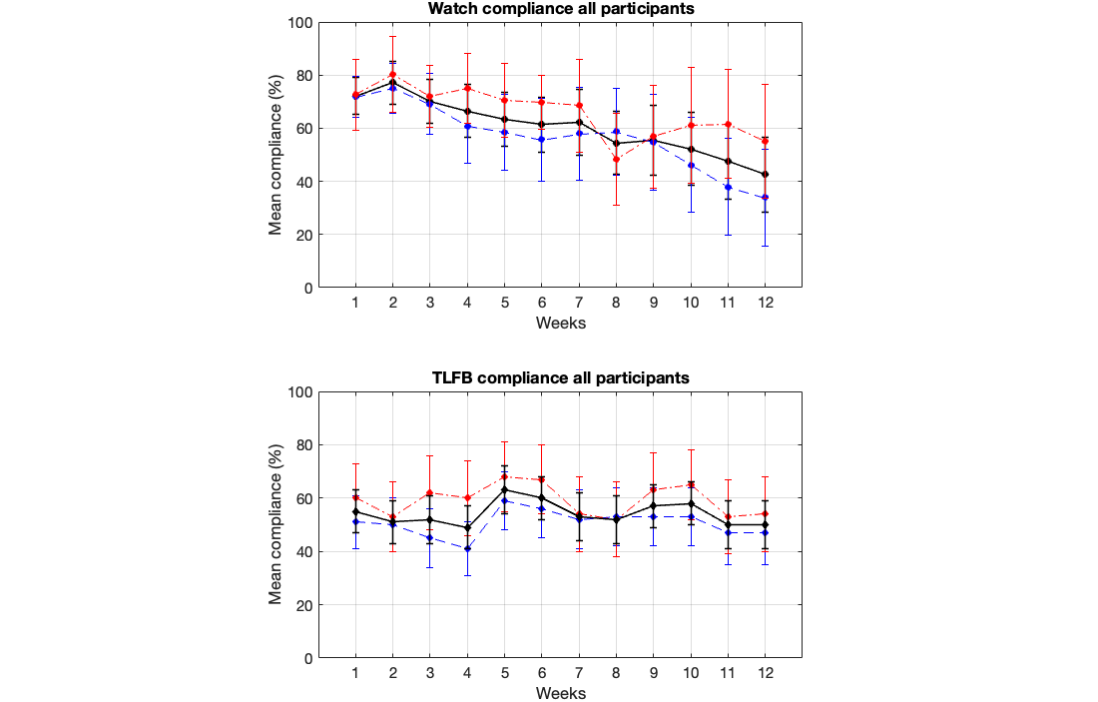
Smartwatch microinteraction-based ecological momentary assessment (top) and timeline followback (TLFB; bottom) compliance rates (mean and 95%CI) across all participants who started the study, for all (black solid line), high-SEP (red dotted dashed line), and low-SEP (blue dashed line) participants. SEP: socioeconomic position.

**Figure 4:**
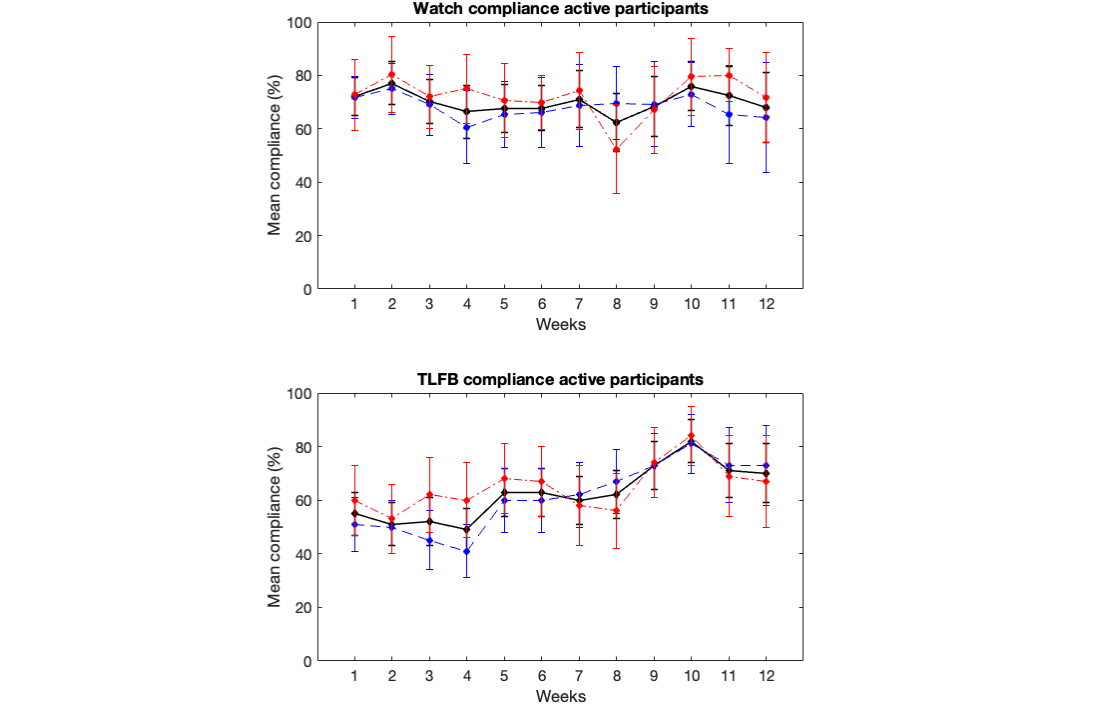
Smartwatch microinteraction-based ecological momentary assessment (top) and timeline followback (TLFB; bottom) compliance rates (mean and 95%CI) across all participants still active in the study for all (black solid line), high-SEP (red dotted dashed line), and low-SEP (blue dashed line) participants. SEP: socioeconomic position.

**Figure 5:**
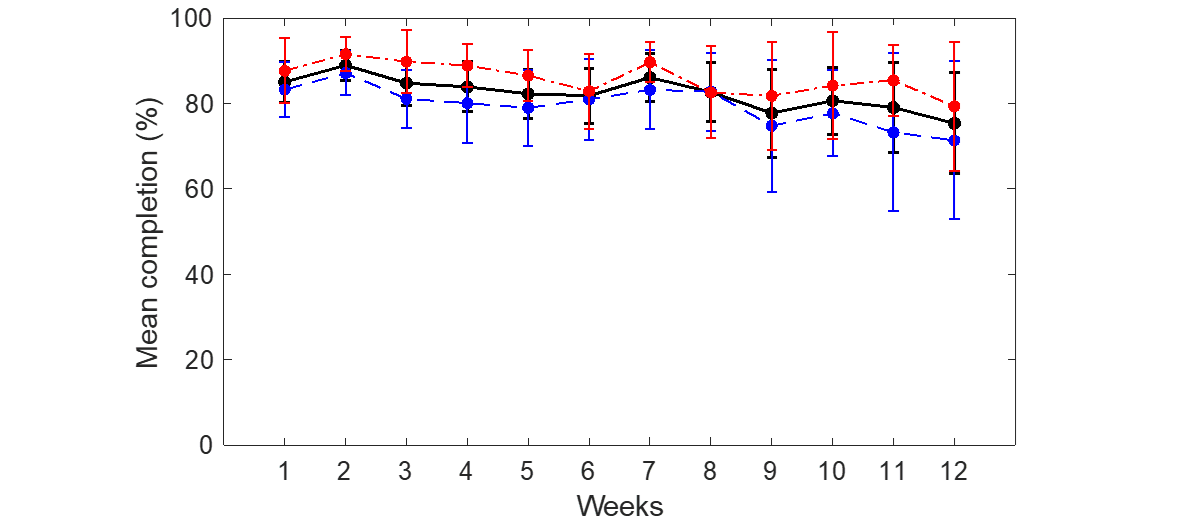
Smartwatch microinteraction-based ecological momentary assessment completion rate (mean and 95%CI) for all (black solid line), high-SEP (red dotted dashed line), and low-SEP (blue dashed line) participants. SEP: socioeconomic position.

**Figure 6:**
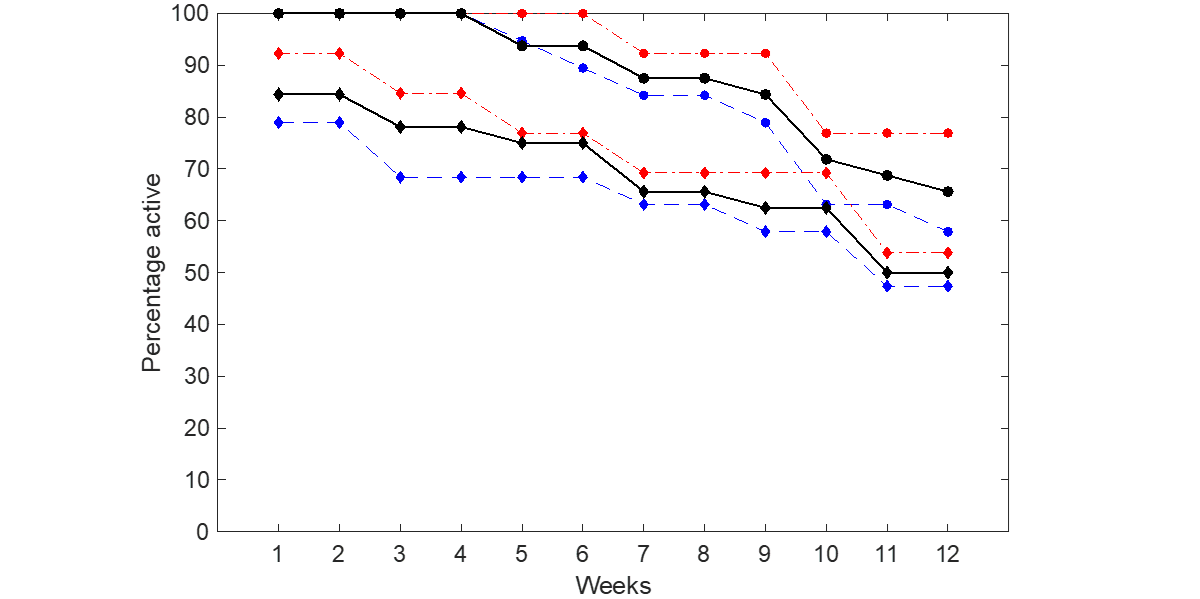
Participant attrition for smartwatch microinteraction-based ecological momentary assessment (circles) and online timeline followback (diamonds) for all (black solid line), high-SEP (red dotted dashed line), and low-SEP (blue dashed line) participants. SEP: socioeconomic position.

Figure 3 presents the compliance rate for all participants who commenced the study using the smartwatch μEMA and online TLFB methods. Figure 4 illustrates the compliance rate for participants who remained active in the study (ie, those who had not yet dropped out) for both methods. Figure 5 displays the completion rate for all active participants using the smartwatch μEMA method (not applicable to TLFB, as explained above). Figure 6 depicts the participant attrition rate, representing the percentage of participants still actively engaged in the study, for both smartwatch μEMA and online TLFB methods.

### Qualitative Feedback

All participants completed interview questions regarding both the online and smartwatch methods for data collection. Participants’ responses to the questions “How would you rate your experience of using the smartwatch μEMA/online TLFB from 1 (did not like) to 10 (really liked)?,” “How willing would you be to use the smartwatch μEMA/online TLFB again in another study from 1 (would not) to 10 (really like to)?,” and “How long would you be willing to use it for (in months)?” are presented in Table 1.

Themes identified from participants’ responses to the question “What things did you like and dislike about using the smartwatch system?” are presented in Table 2, grouped by SEP. The equivalent themes for the online TLFB system are shown in Multimedia Appendix 3. Table 2 also includes participants’ responses to the questions “How did you feel about having to charge the smartwatch every night?,” “Did you pair the smartwatch with any smartphones during the study?,” and “Did you use any of the other functions on the smartwatch during the study (eg, step counter)?”

Distinct themes identified from participants’ responses to the questions “How would you feel if the smartwatch system used data from its GPS location sensor to determine the best time to ask you questions (eg, to avoid prompting during work)?” and “How would you feel if the smartwatch system used data from its motion sensors to determine the best time to ask you questions (eg, to avoid prompting while driving)?” are presented in Table 3.

Because of a technical issue with the questionnaire delivery, 6 participants (2 high SEP [1 female] and 4 low SEP [2 female]) were not asked the questions about smartwatch charging, pairing and other functions, or about the potential use of GPS and motion sensors.

**Table 1:** Participants’ ratings of the extent to which they liked using, would be willing to use it again, and for how long, for the smartwatch μEMA^a^ (Watch) and online (TLFB^b^) methods.

Participants’ ratings of smartwatch µEMA/TLFB	All SEP^c^, median (IQR)		Low SEP, median (IQR)			High SEP, median (IQR)	
	Watch	TLFB	Watch	TLFB	Watch		TLFB
Experience of using	8 (6-8)	7 (6-8)	8 (6-8)	7 (5-9)	8 (7-8)		8 (5-8)
Willing to use again	10 (7-10)	8 (7-10)	10 (7-10)	9 (7-10)	9 (6.5-10)		8 (6-10)
How long for (months)?	3 (3-12)	4 (3-12)	6 (3-12)	6 (3-12)	3 (2-5)		3 (2-5)

^a^μEMA: microinteraction-based ecological momentary assessment.

^b^TLFB: timeline followback.

^c^SEP: socioeconomic position.

**Table 2:** Participants’ likes and dislikes of the smartwatch μEMA^a^ method, attitudes to daily charging, attempts to pair with phone, and other watch features used, for low- and high-SEP^b^ participants.

Participants’ likes/dislikes of smartwatch µEMA			Low SEP, n/N (%)		High SEP, n/N (%)
**Watch liked**					
	Quick and easy	9/19 (47)		6/13 (46)	
	Being prompted	1/19 (5)		4/13 (31)	
	Having a watch	0/19 (0)		2/13 (15)	
	Liked the smartwatch	2/19 (10)		2/13 (15)	
	Additional smartwatch features	2/19 (10)		0/13 (0)	
	No need for extra device	0/19 (0)		2/13 (15)	
	Always to hand	4/19 (21)		2/13 (15)	
**Watch disliked**					
	Timing/frequency of prompts	4/19 (21)		3/13 (23)	
	Technical issues	3/19 (16)		4/13 (31)	
	Battery life	4/19 (21)		2/13 (15)	
	Watch too big	3/19 (16)		2/13 (15)	
	Unsure how to handle missing drinks	1/19 (5)		2/13 (15)	
	Already have a watch/smartwatch	2/19 (10)		1/13 (8)	
**Attitude to needing to charge every night**					
	No issues	11/15 (73)		8/11 (73)	
	Frustrating	4/15 (27)		2/11 (18)	
**Attempted to pair with a smartphone**					
	Did not attempt	13/15 (87)		9/11 (82)	
	Attempted but failed	2/15 (13)		0/11 (0)	
	Attempted and succeeded	0/15 (0)		2/11 (18)	
**Other watch features used**					
	None	11/15 (73)		5/11 (45)	
	Step counter	3/15 (20)		3/11 (27)	
	Timer	1/15 (7)		2/11 (18)	

^a^μEMA: microinteraction-based ecological momentary assessment.

^b^SEP: socioeconomic position.

**Table 3:** Participants’ views on using GPS^a^ data and motion sensor data to optimize smartwatch μEMA^b^, for low- and high-SEP^c^ participants.

Participants’ attitudes toward the use of sensor data		Low SEP (n=15), n (%)	High SEP (n=11), n (%)
**Attitudes to using GPS in the future**			
	No issues	13 (87)	7 (64)
	Depends on context	3 (20)	1 (9)
	OK if consented	0 (0)	1 (9)
	Concerned about battery life	0 (0)	1 (9)
	Scary	2 (13)	0 (0)
	Intrusive	2 (13)	0 (0)
**Attitudes to using motion sensors in the future**			
	No issues	15 (100)	9 (82)
	Concerns about reliability	0 (0)	1 (9)
	Uncomfortable with idea	0 (0)	1 (9)

^a^GPS: global positioning system.

^b^μEMA: microinteraction-based ecological momentary assessment.

^c^SEP: socioeconomic position.

### Missing Data

The rates of missingness for the smartwatch μEMA method among participants still active in the study, stratified by all, low-, and high-SEP groups, are shown for each day of the week in Table 4. Considerable variability was observed across the 3 groups, with overlapping CIs for all days.

The rates of missingness for the smartwatch μEMA method among participants still active in the study, stratified by all, low-, and high-SEP groups, are shown for epochs within days in Table 5. Considerable variability was observed within each group, with overlapping CIs across epochs. However, there was notably less overlap when comparing the first four epochs (12:00–14:00, 14:00–16:00, 16:00–18:00, 18:00–20:00) with the final epoch (20:00–22:00), which had the highest level of missing data.

**Table 4:** Percentage missingness by day of the week for all, high-, and low-SEP^a^ groups (mean and 95% CI).

Weekday	All participants (n=32), mean (95% CI)	High SEP (n=13), mean (95% CI)	Low SEP (n=19), mean (95% CI)
Monday	28.3 (21.2-35.5)	23.8 (14.4-33.3)	31.4(20.8-42.0)
Tuesday	25.6 (18.8-32.4)	24.9 (15.9-33.9)	26.1 (15.8-36.5)
Wednesday	25.0 (19.4-30.6)	23.6 (14.0-33.2)	26.0 (18.5-33.4)
Thursday	26.5 (21.1-31.2)	24.2 (15.3-33.1)	28.1 (20.6-35.5)
Friday	25.2 (19.1-31.2)	24.9 (14.6-35.1)	25.3 (17.0-33.7)
Saturday	28.0 (21.3-34.6)	25.8 (15.0-36.6)	29.5 (20.2-38.8)
Sunday	28.2 (21.0-35.4)	29.6 (18.2-41.0)	27.2 (17.0-37.4)

^a^SEP: socioeconomic position.

**Table 5:** Percentage missingness by 2-hour epoch for all, high-, and low-SEP^a^ groups.

Epoch	All participants (n=32), mean (95% CI)	High SEP (n=13), mean (95% CI)	Low SEP (n=19), mean (95% CI)
12:00-14:00	26.7 (20.4-32.9)	26.1 (17.3-34.9)	27.0 (17.6-36.4)
14:00-16:00	23.6 (17.7-29.5)	22.2 (13.7-30.8)	24.5 (15.9-33.2)
16:00-18:00	24.7 (18.5-30.8)	23.1 (14.1-32.0)	25.8 (16.8-34.7)
18:00-20:00	26.0 (20.4-31.5)	24.2 (15.5-32.8)	27.2 (19.3-35.1)
20:00-22:00	32.5 (25.5-40.0)	30.7 (20.1-41.2)	33.8 (23.5-44.0)

^a^SEP: socioeconomic position.

### Comparison of Levels of Alcohol Consumed Recorded Using the Two Methods

In the high-SEP group:

- 8 of the 13 (62%) participants had higher levels of alcohol units recorded using smartwatch μEMA than TLFB (percentage increase from TLFB: +44, +63, +27, +34, +76, +19, +2, and +71).
- 4 of the 13 (31%) had lower levels of alcohol recorded using smartwatch μEMA than TLFB (percentage decrease from TLFB: –11, –42, –10, and –50).
- 1 of the 13 (8%) did not have sufficient data for a comparison.

In the low-SEP group:

- 10 of the 19 (53%) participants had higher levels of alcohol units recorded using smartwatch μEMA than TLFB (percentage increase from TLFB: +42, +47, +18, +217, +10, +126, +33, +45, +12, and +15).
- 5 of the 19 (26%) had lower levels of alcohol recorded using smartwatch μEMA than TLFB (percentage decrease from TLFB: –6, –15, –61, –4, and –18).
- 4 of the 19 (21%) did not have sufficient data for a comparison.

## Discussion

### Principal Findings

#### Comparing Smartwatch μEMA and Online TLFB Engagement Metrics

Using the most stringent measure of engagement (compliance rate for all participants who started the study), compliance with the smartwatch μEMA method gradually declined over the 12 weeks, from approximately 70% in week 1 to around 45% in week 12. By contrast, compliance with the online TLFB method remained relatively stable at approximately 50% throughout the study. Based on this metric, the smartwatch μEMA method outperformed the online TLFB method until around week 9, after which their performance was broadly similar.

Comparing the compliance rates for all participants with those for participants still active in the study, and considering the attrition rate, it is evident that attrition significantly contributed to the observed decline in compliance among all participants over time. When focusing solely on participants still active in the study (ie, removing the effects of attrition), compliance with the smartwatch μEMA method remained relatively stable at around 70% throughout the 12 weeks. By contrast, compliance with the online TLFB method increased from approximately 50% in week 1 to around 80% in week 10, before declining to 70% in week 12. As before, the performance of the smartwatch μEMA method surpassed that of the online TLFB method until approximately week 9, after which only minor differences were observed between them. Notably, compliance with the online TLFB method increased during the first 10 weeks of the study. While no definitive explanation can be provided, one possible contributing factor is that COVID-19 lockdowns may have resulted in participants spending more time at home during certain periods, potentially improving ease of access to the online TLFB system. By contrast, access to the smartwatch μEMA method would not have been affected to the same extent, as it was specifically designed to be accessible regardless of location. Consequently, a similar increase in compliance would not be expected for this method.

Turning to the completion rate of the smartwatch μEMA method (not applicable to the online TLFB method, as previously discussed), this remained broadly stable at approximately 85% for the first 8 weeks, before declining to around 75% over the final 4 weeks. This suggests that, when the μEMA application functions correctly and the smartwatch device remains charged to ensure prompt delivery, participants exhibit a strong propensity to respond. The slight decline over the 12-week period indicates that longer data collection periods may still be feasible while maintaining acceptable completion rates using this method.

#### Comparing Engagement Metrics for Low- and High-SEP Groups

Although there is considerable variation within groups, and the differences between groups are generally modest (ranging from approximately 5% to 20%), nearly all point estimates for participant compliance, active participant compliance, completion rate, and attrition suggest lower engagement among low-SEP participants compared with high-SEP participants for both the smartwatch μEMA and online TLFB methods.

#### Patterns of Missing Data

There was considerable variability in all 3 groups (all SEP, low SEP, and high SEP), with overlapping CIs when comparing levels of missing data by days of the week and by 2-hour epochs within each group. The most notable pattern was the lower overlap in CIs observed in the all-participant group when comparing the first 4 epochs (12:00-14:00, 14:00-16:00, 16:00-18:00, and 18:00-20:00) with the final epoch (20:00-22:00), which had the highest level of missing data. Modifications to the μEMA application, such as allowing participants to configure their bedtime individually, may enhance engagement and reduce missing data during this period.

Regarding differences between high- and low-SEP groups, there was considerable overlap in CIs across all days and epochs. However, the point estimates for the proportion of missing data were consistently higher for low-SEP participants than for high-SEP participants across all epochs and on all days except Friday and Sunday.

#### Comparison of Levels of Alcohol Consumed Recorded Using the Two Methods

In both high- and low-SEP groups, approximately twice as many participants recorded higher alcohol consumption using the smartwatch μEMA method compared with the TLFB method over the study period. As this is a feasibility study, it was not powered to detect definitive differences in such comparisons, and these findings should be interpreted with caution. However, given the well-documented issue of underreporting in existing alcohol consumption recording methods [3,4], these preliminary results suggest that further research is warranted to assess whether smartwatch μEMA offers improvements in this area.

#### Qualitative Feedback

Across all participants, both methods were rated highly, with a slight preference for the smartwatch μEMA (8/10) over the online TLFB method (7/10). In the low-SEP group, the smartwatch μEMA received a slightly higher rating (8/10) than the online TLFB method (7/10), while the high-SEP group rated both methods equally (8/10).

For willingness to use the methods again, both scored highly, with all participants showing a preference for the smartwatch μEMA (10/10) over the online TLFB method (8/10). This pattern was consistent across SEP groups, with the high-SEP group rating the smartwatch μEMA slightly higher (9/10 vs 8/10) and the low-SEP group showing a stronger preference (10/10 vs 9/10).

Regarding the duration participants would be willing to use the methods again, online TLFB was rated slightly higher overall (4 months) compared with the smartwatch μEMA (3 months). However, within SEP groups, no differences were observed, with the low SEP group indicating a willingness to use both methods for 6 months and the high-SEP group for 3 months.

For the smartwatch μEMA method, the most appreciated factor was its ease and speed of use. However, participants reported a range of disliked aspects, including the timing and frequency of prompts, technical issues, battery life, and smartwatch size, all of which may have contributed to attrition. Regarding daily charging, 19 out of 26 (73%) participants in both SEP groups had no issues, while 4 out of 15 (27%) in the low-SEP group and 2 out of 11 (18%) in the high-SEP group found it frustrating. Additionally, 22 out of 26 (85%) participants in both groups adhered to study instructions by not pairing the smartwatch with their own smartphone. In the low-SEP group, 2 of the 15 (13%) who attempted to pair the smartwatch with their smartphone were unsuccessful, whereas in the high-SEP group, 2 of the 11 (18%) who attempted were successful. High-SEP participants (6/11, 55%) explored additional smartwatch features more frequently than low-SEP participants (4/15, 27%), with the step counter and timer being the most commonly used features.

Regarding attitudes toward using additional smartwatch sensors for data capture, the low-SEP group was more accepting of both GPS (13/15, 87%, had no concerns vs 7/11, 64%, in the high-SEP group) and motion sensors (15/15, 100% vs 9/11, 82%, in the high-SEP group).

### Comparison With Prior Work

#### Alcohol EMA Studies

Perski et al’s [2] systematic review and meta-analysis of EMA studies on health behaviors reported a median adherence of 84% (IQR 77%-91%) across 175 alcohol EMA studies, with smartphone-based EMA methods showing higher adherence than other approaches (eg, online and paper-and-pen). Notably, no studies included wrist-worn wearable EMA methods. Among the engagement metrics we assessed, the completion rate appeared most comparable to their adherence metric, averaging around 80% over the 12 weeks of our study—placing it within the reported range.

In his review of alcohol EMA methods, Piasecki [27] identified several studies employing high-resolution EMA approaches—primarily smartphone based—that prompted participants for responses throughout the day, with study durations ranging from a few days to 8 weeks. Reported response rates varied between 63% and 90%. While direct comparisons with our engagement metrics are not straightforward, our completion rate (~80%) and active participant compliance rate (~70%) fall within this range. Our all-participant compliance rate started at ~70% but dropped below 60% by week 8, remaining broadly consistent with these studies. However, as our study extended beyond 8 weeks, all-participant compliance declined further to 45% by week 12, suggesting that engagement may decrease more substantially in studies of longer duration.

Howard and Lamb [28] recently employed SMS text message–initiated smartphone-based EMA surveys to study alcohol consumption among undergraduates, reporting high compliance levels for active participants—starting at 89% in week 1 and gradually declining to 70% by week 14. By contrast, our active participant compliance rates were lower, fluctuating around 70% over the 12-week period. While no single factor can be identified as the cause of this difference, potential contributors include the nature of the prompts—the study by Howard and Lamb [28] used audible phone notifications, which may have been more attention grabbing than the smartwatch’s haptic prompts—and differences in reward structures, with their incentives tied to individual survey completion, whereas ours were based on months of study participation. However, prior meta-analyses suggest that variations in reward strategies do not consistently impact engagement levels [11,12].

#### Smartwatch μEMA Studies

To the best of our knowledge, there are currently no broadly agreed benchmarks for compliance, completion, and attrition rates in smartwatch μEMA studies. In a study with a comparable assessment period, Beukenhorst et al [9] used smartwatch μEMA methods to collect self-reported pain data from patients with knee osteoarthritis, administering 4-5 questions per day over 13 weeks. Their attrition rate closely mirrored that of our study, with active participant numbers steadily declining to approximately 70% by week 10. However, by the end of the 13-week period, their attrition rate had dropped further to 42%, whereas ours remained at 65% by week 12. The compliance rate for all participants in the study by Beukenhorst et al [9] was also similar to that observed in our study, starting at approximately 75% before gradually declining to around 50% by week 10 and 45% by week 12. Completion rates showed a comparable pattern, beginning at approximately 85% and remaining around 80% throughout the study duration. Additionally, participants in both studies identified short battery life as a barrier to smartwatch use.

In what may be the longest smartwatch μEMA study to date, Ponnada et al [10] collected self-reported data on physical activity and its effect in young adults over a 12-month period, using smartwatch-based μEMA methods across 24 four-day burst periods. In their preliminary findings at the 6-month mark, the mean overall compliance of μEMA for active participants during burst periods was 67%, comparable to the 70% observed over the 12 weeks of this study. Similarly, their reported mean completion rate of 79% at 6 months aligns with the completion rate in this study, which began at 85% and declined to 75% by week 12.

#### Effects of High and Low SEP

Regarding the impact of SEP on engagement in alcohol EMA studies, Howard and Lamb [28] found no significant differences in the number of students with college-educated parents (a measure similar to the SEP indicator used in this study) across their 4 responder categories (“poor,” “adequate,” “good,” and “super”). However, they noted that the proportion of students with college-educated parents was higher in the “good” and “super” responder categories than in the “poor” category, suggesting that higher SEP may have contributed to increased engagement—consistent with the findings of this study.

Considering the broader impact of SEP on compliance in alcohol studies, Thern and Landberg [29] reported that low-SEP participants had approximately twice as much missing data as high-SEP participants for self-reported factors such as self-rated health, health-related quality of life, and level of social support received. While the current feasibility study is not powered to provide definitive evidence of higher levels of missing data in low-SEP participants, the point estimates observed here align with this pattern.

### Strengths and Limitations

This is the first study to assess the feasibility of using smartwatch-based μEMA methods to collect high-temporal-density data on alcohol consumption over extended periods.

While our smartwatch-based EMA app was largely based on the μEMA methods originally developed by Intille and colleagues [8], a key aspect of true μEMA implementation is the presentation of only 1 question at a time. In our approach, we chained questions to capture details on drink type, size, and consumption context, making it more accurately described as a modified μEMA. However, this modification appeared to have little impact on engagement.

Unfortunately, due to COVID-19, participant recruitment had to be halted early, leading to a reduced sample size and an unequal mix of high- and low-SEP participants. As a result, the generalizability of our findings may be limited compared with studies conducted outside the context of a global pandemic.

Our participants were recruited from a UK birth cohort study [20]. A key advantage of this approach is that future research can explore new methods for integrating high-temporal-density, longitudinal data on alcohol consumption from our smartwatch-based μEMA methods with the extensive phenotypic and genetic data available for these participants. This could enable novel investigations into the impact of alcohol consumption on health. However, a potential limitation is that participants in cohort studies are accustomed to regular assessments, which may have increased their motivation to engage with the study compared to individuals not involved in such cohorts [20].

There is growing recognition that the diverse adherence metrics reported in EMA studies can hinder meaningful comparisons across the literature [10,11]. In this study, we selected adherence metrics primarily to facilitate comparisons with existing smartwatch-based μEMA studies. However, we acknowledge that these metrics do not always allow for direct comparisons with other EMA studies using different methodologies.

### Recommendation for Future Studies

The primary factor affecting adherence in this study appeared to be participant attrition. Feedback from the end-of-study interviews provided insights into potential causes, with some issues—such as smartwatch size and battery life—likely to improve as technology advances. However, other factors, including the timing and frequency of prompts, warrant further investigation to optimize compliance and minimize attrition. Additionally, future studies should consider whether specific contextual factors (eg, study location) influence engagement with μEMA methods. A recent work by Ponnada and colleagues [30] offers a valuable overview of this topic.

The consistency of the differences observed between high- and low-SEP groups in compliance, completion, and attrition suggests the need for further research to quantify these disparities and identify their underlying drivers. This also highlights the importance of incorporating user input from diverse SEP backgrounds during the early stages of system design. Beyond SEP, future research should also consider other demographic factors, such as age, that may influence engagement and the quality of data collected using these methods.

This study has focused on the feasibility of using new smartwatch-based μEMA methods to capture data on alcohol consumption. Future research should also investigate response latency (the time between prompt delivery and response), the validity of the data collected compared with existing methods such as TLFB, and how both feasibility and validity vary across other health-related behaviors beyond alcohol consumption.

As a community developing new EMA methods, it is crucial to adopt metrics that facilitate the widest and most meaningful comparisons of findings. Adherence measurement has become increasingly complex, as new EMA methods leveraging the latest technologies benefit from specific metrics (eg, completion and compliance) that help distinguish between technical limitations and participant engagement. Given these advancements, now may be an opportune time to revisit previous recommendations for standardized reporting in EMA studies [31,32], considering the capabilities of mobile and wearable technology–enabled methods.

### Conclusions

In this study, participants demonstrated higher engagement—reflected in higher compliance and completion rates and lower attrition—when using smartwatch-based μEMA methods compared with online TLFB methods for recording alcohol consumption. These engagement levels were consistent with previous studies employing smartwatch μEMA methods. While participants reported similar experiences with both methods and expressed comparable willingness to use either in the future, they showed a slight preference for the smartwatch μEMA approach over the online TLFB method.

While certain challenges remain—particularly for studies extending over months rather than weeks—our findings highlight the considerable potential of smartwatch μEMA methods in alcohol use research. These methods offer the advantage of capturing high-temporal-density, longitudinal data on alcohol consumption, along with contextual information about drinking events. As with EMA methods more broadly, they are less susceptible to recall and bias errors that affect traditional alcohol consumption reporting methods. However, it is important to acknowledge that social desirability bias remains a significant factor in alcohol reporting, and some level of underreporting may persist despite the use of EMA techniques.

Future studies should investigate factors contributing to participant attrition—the primary driver of reduced adherence—as well as latency issues and the validity of alcohol data captured using these methods. While our study does not provide definitive evidence of engagement differences between high- and low-SEP participants, the consistent pattern of lower engagement observed among low-SEP participants suggests that further research is warranted to explore the existence, magnitude, and underlying causes of such disparities.

## Appendix

Multimedia Appendix 1 AlcoWatch microinteraction-based ecological momentary assessment smartwatch question formatting and sequencing.

Multimedia Appendix 2 Timeline followback diary format.

Multimedia Appendix 3 High- and low-socioeconomic position participants' likes and dislikes of the online timeline followback method.

## Abbreviations

ALSPAC: Avon Longitudinal Study of Parents and Children
EMA: ecological momentary assessment
GPS: global positioning system
SEP: socioeconomic position
TLFB: timeline followback
μEMA: microinteraction-based ecological momentary assessment

## Data Availability

The informed consent obtained from ALSPAC participants does not allow the data to be made freely available through any third-party–maintained public repository. However, data used for this submission can be made available on request to the ALSPAC Executive. The ALSPAC data management plan describes in detail the policy regarding data sharing, which is through a system of managed open access. Full instructions for applying for data access can be found on the ALSPAC researchers’ access web page [33]. The ALSPAC study website contains details of all the data that are available [23].
