# Correction: “Smartwatch-Based Ecological Momentary Assessment for High-Temporal-Density, Longitudinal Measurement of Alcohol Use (AlcoWatch): Feasibility Evaluation”

**DOI:** 10.2196/82194

**Published:** 2025-10-01

**Authors:** Chris Stone, Sally Adams, Robyn E Wootton, Andy Skinner

**Affiliations:** 1 Integrative Cancer Epidemiology Programme Bristol Medical School University of Bristol Bristol United Kingdom; 2 School of Psychological Science University of Bristol Bristol United Kingdom; 3 School of Psychology University of Birmingham Birmingham United Kingdom; 4 Nic Waals Institute Lovisenberg Diaconal Hospital Oslo Norway; 5 Medical Research Council Integrative Epidemiology Unit Bristol Medical School University of Bristol Bristol United Kingdom; 6 PsychGen Centre of Genetic Epidemiology and Mental Health Norwegian Institute of Public Health Oslo Norway

In “Smartwatch-Based Ecological Momentary Assessment for High-Temporal-Density, Longitudinal Measurement of Alcohol Use (AlcoWatch): Feasibility Evaluation” (JMIR Form Res 2025;9:e63184) the authors have updated the email address of the corresponding author.

Corresponding Author Chris Stone’s email address has been updated to: chris.stone@bristol.ac.uk

The correction will appear in the online version of the paper on the JMIR Publications website, together with the publication of this correction notice. Because this was made after submission to PubMed, PubMed Central, and other full-text repositories, the corrected article has also been resubmitted to those repositories.

