# Supplementary figures and images for: Smartwatch-Based Ecological Momentary Assessment for High-Temporal-Density, Longitudinal Measurement of Alcohol Use (AlcoWatch): Feasibility Evaluation
Source: JMIR Form Res. 2025 Mar 25;9:e63184. doi: 10.2196/63184 (PMC11979524; doi:10.2196/63184)

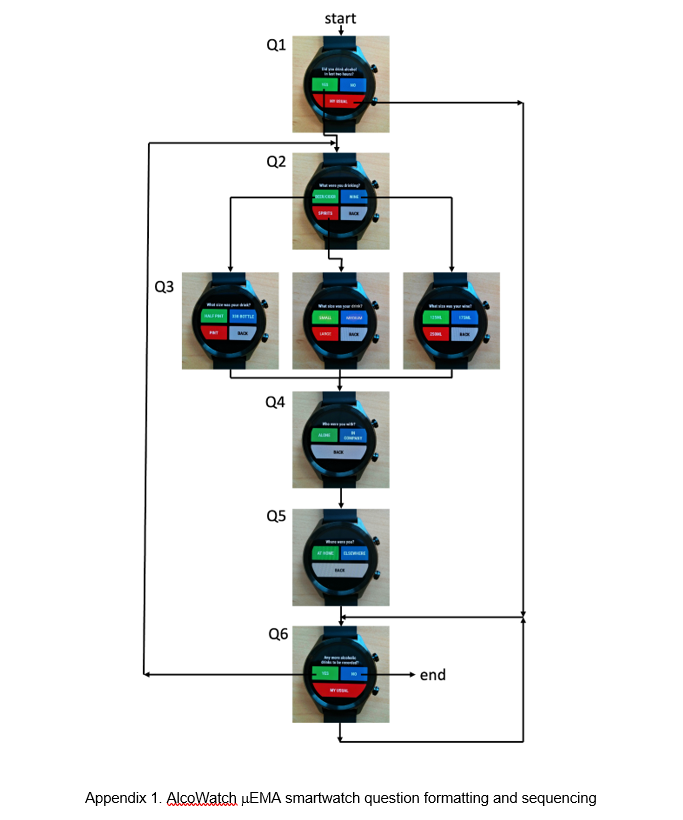

Supplement: Multimedia Appendix 1 [file formative_v9i1e63184_app1.png]

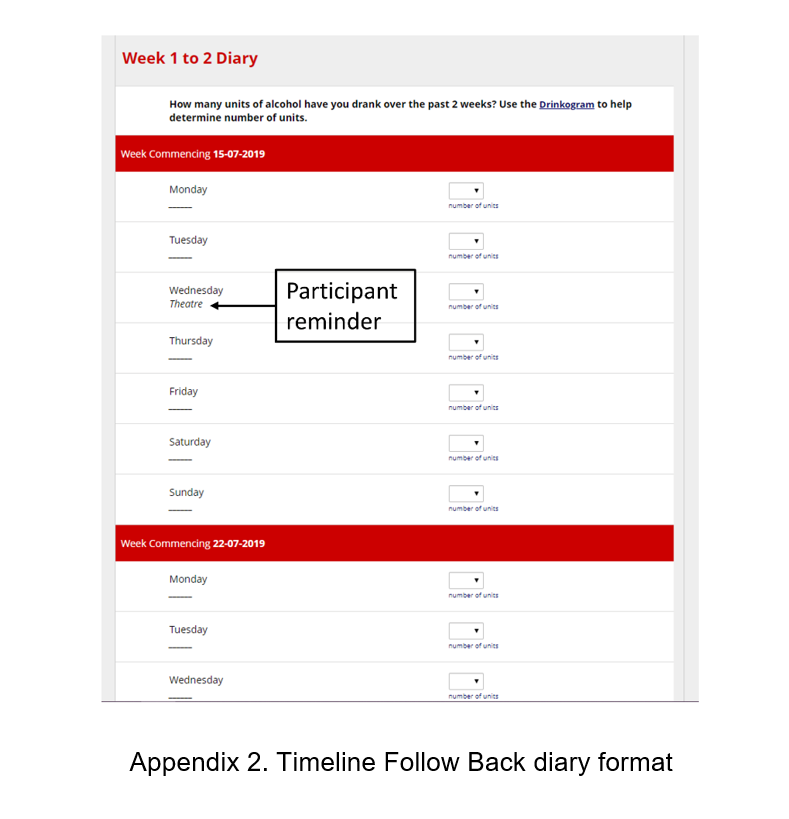

Supplement: Multimedia Appendix 2 [file formative_v9i1e63184_app2.png]

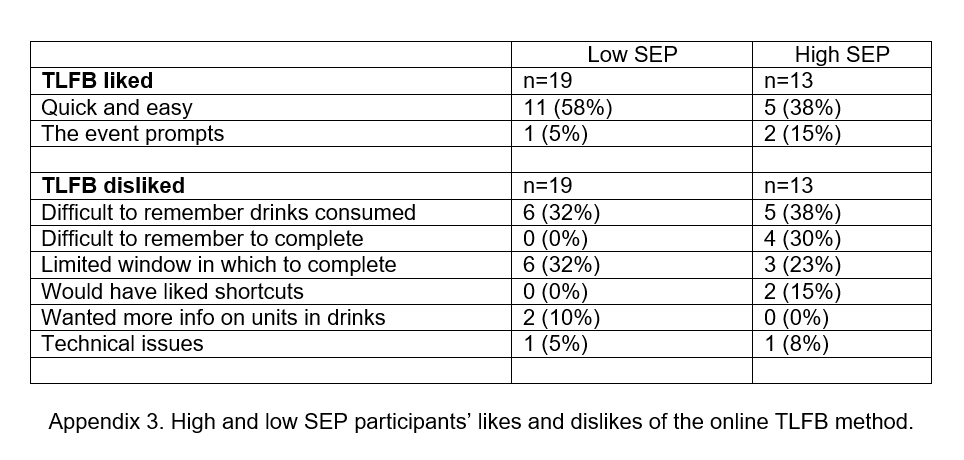

Supplement: Multimedia Appendix 3 [file formative_v9i1e63184_app3.png]
